# Psychiatric Treatment in Pregnancy: A Narrative Review

**DOI:** 10.3390/jcm12144746

**Published:** 2023-07-18

**Authors:** Iga Gruszczyńska-Sińczak, Katarzyna Wachowska, Katarzyna Bliźniewska-Kowalska, Piotr Gałecki

**Affiliations:** Department of Adult Psychiatry, Medical University of Lodz, 91-229 Lodz, Poland

**Keywords:** pregnancy, psychiatric treatment

## Abstract

Pregnancy, childbirth and the puerperium are a series of transformations and huge changes in a woman’s life, which may also be accompanied by various mental problems. Very often, women experiencing mental disorders during this period and their doctors face a decision on safety of treatment. The purpose of the following review was to assess the safety of treatment during pregnancy. Internet scientific database PubMed was searched. There are groups of psychiatric medications contraindicated during pregnancy such as valproates as well as relatively safe ones such as selective serotonin reuptake inhibitors (SSRIs) or antipsychotics. However, in every clinical situation, a decision should be made with caution, based on individual characteristics of patient, severity of disorder and clinical picture.

## 1. Introduction

Pregnancy, childbirth and the puerperium are a series of transformations and huge changes in woman’s life, which may also be accompanied by various mental problems. Hormonal changes and increased stress experienced by women during this period are just some of the factors that increase the risk of mental disorders. The latest ICD-11 classification (International Statistical Classification of Diseases and Related Health Problems, version 11) has developed a new category of mental problems occurring in association with pregnancy, childbirth and the postpartum period, which includes specific mood disorders and psychotic disorders (block L1-6E2) [1]. They are presented in Table 1. Proper diagnosis and treatment of mental health problems occurring during pregnancy are crucial for the lives of both babies and mothers. However, choosing pharmacological treatment might not be easy because drugs beneficial for mother might be harmful for the developing fetus. Therefore, the present paper outlines psychiatric treatment options in a view of their safety during pregnancy.

## 2. Materials and Methods

**Figure 1:**
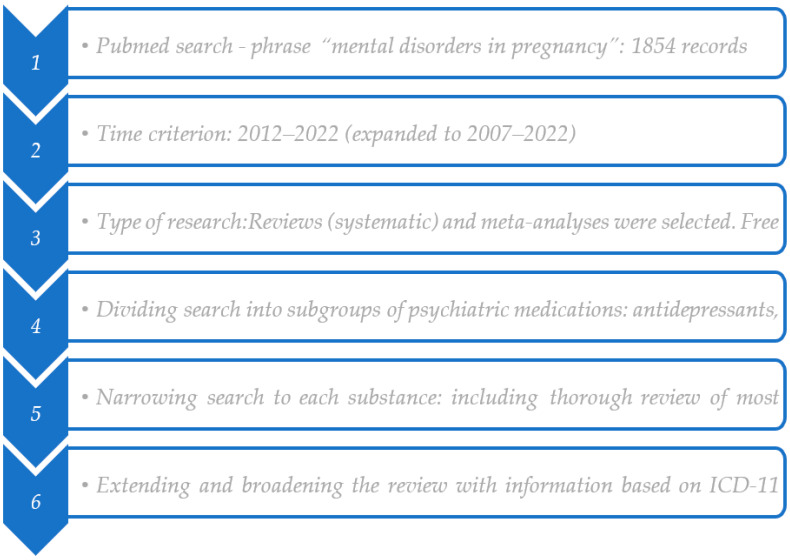
Decision-making schema.

The purpose of the following review was to assess the safety of psychiatric treatment during pregnancy. When preparing the review, authors followed the PRISMA statement checklist. See the decision-making scheme (Figure 1). 

The first step was to search the Internet scientific database PubMed with the phrase “mental disorders in pregnancy”. There were 1854 results.

The next step was restricting the search to each group of medications used in psychiatry.

The third, last level of the review was checking articles on specific drugs (substances) in combining them with a disease entity, narrowing it down to the topic of pregnancy.

Initially, the criterion of 10 years was used, in the period 2012–2022. In the next stage this was extended by reviewing articles published up to 15 years earlier.

Reviews (systematic) and meta-analyses were selected. Free access, full articles were used.

During the final preparation of the article, the information was extended with data from books published over the last five years and classifications: ICD10, ICD11 (International Statistical Classification of Diseases and Related Health Problems, version 10 and 11) and DSM 5 (Diagnostic and Statistical Manual of Mental Disorders, version 5).

## 3. Epidemiology of Mental Problems in Pregnancy and Postpartum

Mental disorders after childbirth may be a one-off episode, the beginning of a new disease or an exacerbation of the disorder that has been treated in the past. Particular attention should be paid to patients who were treated psychiatrically before pregnancy, even if they are in a long-term symptomatic remission. Patients after becoming aware of the pregnancy tend to stop taking drugs overnight, without consulting a doctor, which contributes to a quick recurrence of the disease. According to the estimates of the World Health Organization (WHO), globally, about 10% of pregnant women and 13% of postpartum women experience mental disorders, mainly depression. In developing countries, this percentage may range from 15.6% (in pregnant women) to 19.8% (in postpartum women) [2]. There are survey studies in which the incidence of postpartum depression is estimated at the level of 10% to 30%. Based on the information on the incidence of childbirth depression in Poland, the level of 13% was estimated in accordance with WHO data [3]. A very common phenomenon observed after childbirth is “postpartum blues”. It has been estimated that it occurs in as many as 40–85% of women. It is characterized by irritability, emotional lability, tearfulness, restlessness, sleep and concentration disorders. However, the presenting symptoms do not allow for the diagnosis of depression. This process resolves spontaneously and does not affect professional and social functioning [4,5].

According to the World Health Organization (WHO), in 2030, depression will be the second leading cause of disability in the world. It is also one of the most common complications during pregnancy. The greatest risk of developing the disease falls on the second and third trimesters. The prevalence of pure postpartum depression is estimated at around 13–19%. In addition, 20–50% of postpartum bipolar disorders are diagnosed in patients with previously diagnosed bipolar affective disorder [2]. The incidence of perinatal psychosis is estimated at 0.1–0.25% in the general population. In women who previously suffered from schizophrenia, it is 12%, while in women diagnosed with schizoaffective or bipolar affective disorder before pregnancy, it reaches as much as 72–80%. If a patient experiences psychosis during a previous birth, the risk of recurrence is estimated at 50–90% [5,6,7].

## 4. Etiology of Mental Disorders in the Perinatal Period

Mental disorders in the perinatal period have a multi-causal etiology. Physiological changes occurring in the mother’s body are considered to be one of the causes in genetically susceptible women. These include primarily changes in the endocrine system and the autoimmune system. Therefore, follow-up laboratory tests should be performed to exclude autoimmune thyroiditis. The impact of infectious diseases is to be taken into consideration, too. Drugs used by pregnant women, including glucocorticosteroids and sympathomimetics, are not without significance. Conditions associated with brain dysfunction, such as stroke in the course of eclampsia or pre-eclampsia, and the neoplastic process might also have an impact. Risk factors for the development of depressive disorders in the perinatal period are various. They include socioeconomic factors such as lack of social and partner support, poor marital relations, single motherhood, negative life experiences, lack of work and poor financial situation, lack of experience in childcare, having more than three children and mother’s age below 20. Pregnancy-related factors also play a potential role: unwanted pregnancy, high-risk pregnancy, traumatic experiences in previous pregnancies (miscarriage, abortion, diseases, genetic defects), difficult or traumatic childbirth, multiple births, sleep disorders and sleep deprivation. Other risk factors include history of mental disorders both in the mother and in the family (recurrent depressive disorders, bipolar affective disorder, anxiety disorders, suicide attempts in the past, addiction to psychoactive substances) and the mother’s personality traits (higher level of neuroticism, low self-esteem, anxious personality, anankastic emotional instability). There are others that might be very important, such as emotional abuse in the relationship, poor relationship with the mother, illnesses of the child, poor prenatal care and experiences of violence in childhood [2,4,5,6,7,8,9].

## 5. Diagnostic Criteria

Classifications are sets of features characterizing a given disease, which serve to unify the diagnoses. They are designed to facilitate the diagnostic process. In Europe, the use of the International Classification of Diseases (ICD) International Statistical Classification of Diseases and Related Health Problems has been adopted. Currently, many countries are facing difficulties due to the process of implementation of the eleventh edition. In the United States of America, the classification of the American Psychiatric Association-Diagnostic and Statistical Manual of Mental Disorders, i.e., DSM, currently (since 2013) the fifth edition, is used. Table 2 presents diagnostic criteria differences [1,10,11,12].

## 6. Early Detection and Diagnosis

Screening for postpartum depression (PPD) is recommended as part of the routine care of a pregnant and postpartum patient. The U.S. Preventive Services Task Force (USPSTF) (2016) recommends routine screening of the adult population and pregnant and postpartum women. At the same time, it indicates the need to provide the examined person with access to further care and coordinated treatment [13]. American Psychiatric Association (APA), (2010) and American College of Obstetricians and Gynecologists (ACOG) (2007) recommend routine EPDS screening. The Edinburgh Postnatal Depression Scale Consists of 10 questions to measure a woman’s mood in the postpartum period in the last 7 days [13]. Each question has four possible answers (0–3). The maximum score that can be obtained is 30 points. Scores above 12 points should be evaluated clinically. A score of 10 is also thought to suggest emotional problems in obstetrics. Performed 6 weeks after delivery, it is effective in reducing the risk of PPD. Another scale is the Beck Depression Inventory (BDI). It enables the patient’s self-assessment of her depressive symptoms. It is filled in by the respondent. It can cover different periods: a day, a week or a month. It contains 21 groups of statements (A-U) scored from 0 to 3. It is a standardized tool for assessing the severity of depression and distinguishing between healthy and sick people. It can determine the frequency of diagnosing depression, recognize the severity of an episode and assess the effectiveness of pharmacotherapy. Other scales include the questionnaire of risk factors for mental disorders in pregnancy, questionnaire of risk factors for mental disorders after childbirth, Postpartum Depression Predictors Inventory (PDPI), antepartum questionnaire (APQ) and Postpartum Checklist Bromley Postnatal Depression Scale (BPDS) 4 for PPD during a gynecological check-up 4–6 weeks after delivery. The pregnant patient should be educated about the possible complications of PPD. Patients with baby blues require specific monitoring and assessment of depressive symptoms [14]. The American Academy of Pediatrics Bright Futures (AAP) (2010) highlights the key role of a pediatrician in ensuring health of the whole family because mother’s mental problems directly affect the child’s development. Guidelines recommend that pediatricians routinely assess for depression in women who visit their children. The American College of Nurse-Midwives (2003) recommends screening for PPD as part of routine prenatal and postnatal care. The National Institute for Health and Care Excellence (NICE) (2014) recommends routine assessment of depressive symptoms in every woman in the perinatal period using standard screening tools. This examination should be performed at least twice (at the first visit during pregnancy and in the first year after delivery). NICE recommends a set of two initial ”Whooley questions”: “Have you felt sad, depressed or hopeless in the last month?” “Did you notice a decrease in enjoyment of your activities in the last month?” If a woman answers positively to either of the two questions or is at risk for mental illness, or if her clinical history is indicative of depression, a full assessment of her mental status using the EPDS or Patient Health Questionnaire (PHQ-9) screening tools or referral for further treatment (family doctor or specialist psychiatrist) is recommended. It also recommends asking the woman about her history of alcohol and drug addiction [15]. The Scottish Intercollegiate Guidelines Network (SIGN) (2012) recommends a minimum of three assessments (at the first pregnancy visit, 4–6 weeks and 3–4 months after delivery). Moreover, it is advisable to include a history of affective disorders and, in the case of a positive history, a screening test for depression at each visit. Like NICE, SIGN recommends a set of two initial screening questions and, if further assessment is needed, using the EPDS scale [16]. The Beyond Blue Guidelines for Primary Care Health Professionals (2011) recommend using the EPDS to screen all pregnant and postpartum women for depression as part of their assessment of depressive and anxiety symptoms. Screening should be carried out between 6 and 12 weeks postpartum at a follow-up visit. A score of 13 or more can be interpreted as postnatal depression [17]. The American Psychiatric Association (APA) recommends non-pharmacological interventions including cognitive behavioral therapy (CBT) and interpersonal therapy. The risk of potential exposure of the child to the drug should be considered when deciding on the use of pharmacological treatment during breastfeeding [18]. The National Institute for Health and Care Excellence (NICE) (2014) recommends a step-by-step approach to the treatment model (this does not apply to women with a severe illness episode, who should be immediately referred to specialist psychiatric care). Mild to moderate PPD can be successfully treated at the primary care level. NICE also emphasizes that a comprehensive visit and treatment plan should be prepared for women already diagnosed with a mental illness [15]. The Scottish Intercollegiate Guidelines Network (SIGN), (2012) states that CBT should be considered in women with mild to moderate PPD. Both SSRIs and tricyclic antidepressants may be recommended for the treatment of moderate to severe postpartum depressive episodes after careful assessment of the risk to the breastfed infant [16]. The Beyond Blue Guidelines for Primary Care Health Professionals (2011) recommended non-pharmacological interventions, which include psychological support, CBT, interpersonal therapy and psychodynamic therapy. The decision to use pharmacological treatment during breastfeeding should take into account the risks of potential exposure of the child to the drug [17]. It should be emphasized that the treatment of mental disorders during pregnancy, childbirth and the postpartum period is extremely important. Otherwise, it may have negative consequences for both the mother and the developing fetus. Negative consequences may vary, depending on the individual characteristics of the patient. However, major and most alarming examples of potential dangers for mother are suicide risk, poor self-care, worse co-operation with obstetrician and reduced interest in child’s well-being from the mother’s perspective. On the other hand, the mother’s distress and depression may affect the child through abnormal hormonal levels, poorer nutrition, fatigue or unhealthy behaviors such as substance use.

## 7. Psychopharmacology in Pregnancy

The following paragraphs are focused on a review of the main groups of psychiatric medications and most prominent examples of each. All of them have been specifically considered in terms of safety in pregnancy.

### 7.1. General Rules of Psychiatric Treatment during Pregnancy

**Figure 2:**
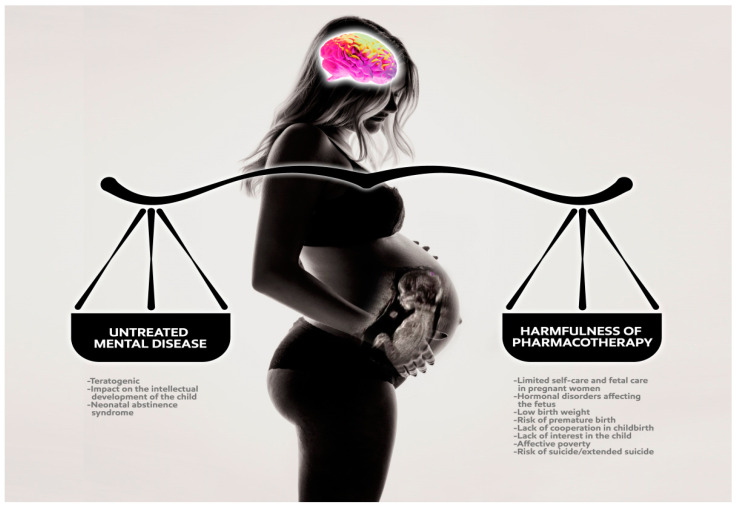
Balancing the risks of potential harmfulness of pharmacotherapy in pregnancy vs. consequences of untreated mental disorder.

Multidisciplinary cooperation between the gynecologist, obstetrician, psychiatrist, psychologist and psychotherapist is necessary. This might help to avoid hospitalization. Each interaction should be documented in the medical history. The decision taking into account the balance of benefits and losses for the mother and child resulting from pharmacotherapy during pregnancy must be consciously made with the patient. The treatment must always be based on the latest data in the literature, including non-pharmacological methods. The patient should properly supplement her diet by taking, for example, folic acid. When we decide to start the drug or when its discontinuation is not possible, we choose the lowest effective dose. We use as few substances as possible and monitor the therapy continuously. The teratogenic risk is highest in the first trimester. If possible, it is reasonable to withhold pharmacotherapy until the second and even the third trimester. Any medication should be avoided in the first trimester (maximum teratogenic potential: days 17–60; weeks 2–9). The negative effect of drugs on intellectual development and their impact on childbirth is important mainly in the second and third trimesters. The main recommendation is to use the lowest effective dose while monitoring the effects of the drug (positive and negative). Frequently, the risk of recurrence is greater than the risk of fetal damage, and a higher dose is warranted. It is important that drug pharmacokinetics may be variable during pregnancy, and attention should be paid to the need for dose adjustments. When symptoms of mental state disorder are excessive, hospitalization should be considered. For some drugs (SSRIs, serotonin norepinephrine reuptake inhibitors (SNRIs), tricyclic antidepressants (TCAs), benzodiazepines (BDZs)), withdrawal effects were reported in newborns, specifically neonatal abstinence syndrome (NAS). If possible, pharmacotherapy should be gradually discontinued or reduced several weeks before the planned date of delivery. If this is not possible, it is recommended to alternately feed the newborn with the mother’s and formula milk. The choice of the drug is dictated by the lack of data related to the risk to the fetus, rather than by unequivocal, confirmed safety. In the past, the U.S. Food and Drug Administration (FDA) classification recommended safety assessment of the used drugs. Depending on the teratogenic potential, drugs were assigned to five categories (A, B, C, D, X). Currently, the FDA has withdrawn the above division, suggesting the use of the characteristics of medicinal products, the Pregnancy and Lactation Labeling Rule (PLLR). Therapeutic decisions can be difficult due to the lack of data and sometimes their contradiction (Figure 2). The effect of many psychotropic drugs on immediate and long-term outcomes in infants remains unclear, but in many cases it is better to treat than risk complications [19].

### 7.2. Antidepressants

SSRIs are the most commonly used antidepressants. There is no specific drug dedicated to use in pregnancy, but attention has been particularly paid to citalopram, escitalopram and sertraline. It is known, however, that paroxetine is absolutely contraindicated due to the increased risk of congenital heart defects in the fetus. There is no strong evidence that other drugs in this group increase the risk of malformations and birth defects [20]. SSRIs are not recommended in the first trimester and have been reported to cause congenital heart disease, persistent pulmonary hypertension, premature suture and fibrous closure of the skull and umbilical ring hernia. However, in general, they have not been shown to have certain harmful effects on the fetus. Interestingly, mothers treated with serotonin reuptake inhibitors have an increased risk for bleeding during delivery. They also increase the risk of pregnancy-induced hypertension or pre-eclampsia. After delivery, the newborn may show temporary irritability or sedation. Low birth weight is mentioned in the research, but it is worth noting that depression alone during pregnancy increases the risk of low birth weight. One recent study showed a small risk of premature birth when SSRIs were taken in the first trimester. However, an increased risk of having a child with autism spectrum disorder and attention deficit hyperactivity disorder (ADHD) was not demonstrated [21,22,23]. SSRIs and SNRIs used in the third trimester may lead to complications resulting in prolonged hospitalization; respiratory support and tube feeding are necessary. Drug toxicity or withdrawal symptoms (respiratory failure, cyanosis, convulsions, thermoregulation disorders, feeding difficulties, vomiting, drop in glucose level, weakness or increased tension, tremors, increased reflexes, inconsolable crying) are considered to be the cause [2].

Regarding TCAs, we have relatively few data on this group of drugs. These substances are known to cross the placenta. Clomipramine is completely contraindicated because it causes fetal cardiovascular malformations [6,24].

#### A Review of Research on Antidepressants

Fluoxetine, according to Shan-Yan Gao et al. (2017), is a drug which may cause cardiovascular defects in infants when used in the first trimester of pregnancy. The results were obtained as a result of a systematic search of the PubMed and Web of Science databases. A total of 1918 pre-identified articles were qualified, from which 16 cohort studies were identified [21]. In studies on the effects of sertraline on the fetus, Line Kolding et al. (2019) concluded that it did not show an increased effect on fetal heart disorders compared to pregnancies of women not using this drug. The tricuspid annular plane systolic excursion (TAPSE), mitral annular plane systolic excursion (MAPSE), myocardial perfusion imaging (MPI) and E/A (perception of the external appearance of mitral inflow and mitral inflow during atrial systole) indices were used in the study. Forty-four women were enrolled, fifteen of whom were using sertraline and the rest were not exposed to pharmacotherapy [22]. This issue was also explored by Anick Bérard et al. (2015), who examined 18,493 pregnancies using a cohort study. They showed that sertraline had an effect on the increased risk of atrioventricular defects and craniosynostosis. According to the authors of the article, other SSRIs used by pregnant women increased the risk of craniosynostosis and musculoskeletal defects. The study enrolled 18,493 women who received medication in the first trimester of pregnancy. Of these, 366 were exposed to sertraline, 1963 to other SSRIs and 1296 to non-SSRI antidepressants [23]. According to Kayla N. Anderson et al. (2020), there were links between the use of antidepressants and the occurrence of birth defects. The most commonly used antidepressants in pregnancy were sertraline, fluoxetine, paroxetine, citalopram, escitalopram, venlafaxine and bupropion. In the SSRI group, the highest rates of birth defects occurred with paroxetine and fluoxetine, followed by citalopram and sertraline less frequently, and no significant increase in risk was found with escitalopram. Venlafaxine is reported to be associated with the highest number of birth defects, although this issue requires further study. There is also an adverse effect of bupropion on the formation of birth defects. The study involved 30,630 mothers of infants diagnosed with congenital defects and 11,478 mothers from the control group [20]. In a cohort study, Krista F. Huybrechts et al. (2014) showed no significant increase in the risk of congenital heart defects when using antidepressants in the first trimester of pregnancy. The study involved 949,504 pregnant women; in the first trimester of pregnancy, a total of 64,389 women used antidepressants. The association between fetal heart defects and antidepressant use declined as an increasing number of additional factors were taken into account. No significant association was found between the use of paroxetine and right ventricular occlusion or between the use of sertraline and the occurrence of ventricular septal defects [25]. Safety of sertraline has also been presented by Cuomo (2018) and Kolding (2023) as well as escitalopram by Bellantuono (2012; 2013) [26,27,28,29]. TCAs have been discussed by Gentile (2014) [30]. Vitale (2016), Gao (2018), Womersley (2017) and Nielsen (2012) also summarize the safety of antidepressive treatment in pregnancy [31,32,33,34] (Table 3).

### 7.3. Mood Stabilizers

#### 7.3.1. Valproates

Use of valproates carries a very high risk of teratogenesis, reaching 10%. It is known that the higher the dose, the greater the harm to the fetus. Valproates may have an adverse effect on the mental and physical development of children, i.e., they tend to start to talk and walk later and have poor mental skills, memory problems and lower intelligence quotient (IQ). Studies also indicate an increased risk of autism spectrum disorders and attention deficit hyperactivity disorder (ADHD). Usage of valproic acid during later stages of pregnancy might lead to neonatal toxicity syndrome (irritability, feeding problems, muscle tone disorders, toxic liver failure, coagulopathies, hypoglycemia). According to the guidelines of the European Medicines Agency (EMA) and the Office for Registration of Medicinal Products, Medical Devices and Biocidal Products established in 2018, the use of valproic acid is contraindicated during pregnancy. When it turns out that a patient treated with valproate is pregnant, modification of pharmacotherapy should be started as soon as possible, gradually discontinuing the substance harmful to the fetus, including a lower-risk mood stabilizer (Table 4) [35,36,37,38,39]. It is important to remember the teratogenic effect of valproate. This medication is contraindicated in pregnancy and should be used with caution in fertile women (contraceptives should be administered) [24].

#### 7.3.2. Carbamazepine

The effect of carbamazepine on teratogenesis has been questioned in new research. The risk of birth defects with carbamazepine monotherapy is 3–6% on a dose-adjusted basis. A dose above 700 mg increases the risk from 4.5% to 7.2%. The most common birth defect is spina bifida. As a preventive measure, it is recommended that pregnant women take folic acid. In addition, there may also be a cleft palate, cardiovascular disorders, urinary tract disorders and underdevelopment of nails. Possible craniofacial malformations are short nose, elongated philtrum, epicanthal wrinkles, hypertelorism and ascending palpebral fissures. In later pregnancy, it may be associated with hepatotoxicity, microcephaly, growth retardation, premature birth, vitamin K deficiency and coagulopathy. It is difficult to determine the absolute risk of adverse neurodevelopmental effects in the fetus. The results of the research indicate the possibility of neurodevelopmental delay and a decrease in IQ. When it is not possible to avoid the use of valproates or carbamazepine during pregnancy, supplementation with 1 mg of folic acid daily and the addition of vitamin K in the third trimester are recommended [24,35,36,37,38,39].

#### 7.3.3. Lithium Carbonate

Lithium carbonate is the oldest and best-known drug from the group of mood stabilizers. It is also considered the most effective of this group. Unfortunately, its use carries a high risk to the fetus. Table 5 presents the effects of lithium carbonate use depending on the period of pregnancy. Currently, there is no clear relationship between the use of lithium and the neurodevelopmental and neurobehavioral effects in children. Abrupt discontinuation of the drug is associated with a high risk of maternal relapse (50%). Changes in the woman’s body (changes in total body water, plasma volume and glomerular filtration) should be taken into account when using lithium. There is an increase in clearance and a decrease in lithium concentration. The concentration of the drug decreases in the first and second trimesters but increases in the third trimester and perinatal period. The increase in the level of lithium in the body is also affected by conditions such as vomiting or pre-eclampsia; therefore, it is crucial to monitor the fluid balance. It is necessary to constantly monitor the therapy, initially every 4 weeks and every week after the 34th week [24,36,37,38,39,40].

#### 7.3.4. Lamotrigine

Lamotrigine has a lower risk of malformations compared to valproic acid, carbamazepine and lithium. When lamotrigine is used alone, the risk of major birth defects is estimated at 2–3%, which is the range of birth anomalies in the general population. The increase in risk is proportional to the dose. The risk of serious malformations when the dose of 325 mg per day is exceeded increases from 2.5% to 4.3%. There are possible increases in the risk of oral clefts, hypospadias and gastrointestinal defects when exposed in the first trimester. Folic acid supplementation has the effect of reducing the effectiveness of lamotrigine. There is no evidence of problems with emotional, behavioral or cognitive functioning in children due to the use of lamotrigine during pregnancy. Glucuronidation is the process by which lamotrigine is metabolized in the liver. The glucuronidation process is induced by estrogen, leading to increased drug clearance. In the second and third trimester, the dose of the drug should be increased to 315% of the initial dose to maintain the therapeutic concentration of the drug. As the clearance of lamotrigine decreases within a few days after delivery, care should be taken to reduce the dose to prevent intoxication [24,35,36,37,38,39].

#### 7.3.5. A Review of Research on Mood Stabilizers

According to Yusuf Cem Kaplan et al. (2021) using the UK registry of Campbell et al., the risk of serious fetal defects increases in proportion to the increase in the dose of carbamazepine. However, comparing the data from the North American Pregnancy Registry and the Australian Pregnancy Register in the case of carbamazepine monotherapy, no similar relationship was found between the dose used and the risk of defects. Regarding valproates, Yusuf Cem Kaplan et al. (2021) compared the following studies: Samren et al., little significant relationship between the dose of valproate and its harmfulness; Kaneko et al., significant correlation between the dose and the incidence of major malformations (<1000 mg/day); Artama et al., significant negative effect depending on the dose >1500 mg/day; Meador et al., the only antiepileptic drug associated with dose-dependent adverse effects on the fetus (900 mg/day) [35]. It has been noted that, according to the UK Epilepsy and Pregnancy Register, as the dose of valproate increases, the risk of birth defects increases, albeit not significantly. Diav-Citrin et al. saw an eight-fold increase in malformations at doses greater than or equal to 1000 mg/day. Interestingly, no increased risk of defects was noted in this study at doses below 1000 mg/day. The North American Antiepileptic Drug (AED) Pregnancy Registry registered very similar claims for doses >1000 mg/day and below 750 mg/day. Tomsona et al. compared the safety of valproate use in polytherapy and the effect of the dose level, observing an increase in the occurrence of malformations in the case of polytherapy and an increase in the dose used (above 1500 mg/day). Also, according to the European Registry of Antiepileptic Drugs and Pregnancy (EURAP), there was a noticeable relationship between the dose of valproate and the occurrence of birth defects. In the Australian Pregnancy Register analysis, the risk of birth defects becomes statistically significant at a dose of 700 mg of valproate per day. When examining the safety of lamotrigine, Yusuf Cem Kaplan et al. (2021) noted that, according to Morrow et al., the dose significantly affects the development of fetal malformations (above 200 mg). On the other hand, Campbell et al. did not show a statistically significant relationship between the dose of lamotrigine and the increased risk of birth defects. The EURAP study found a statistically significant difference between doses above or below 325 mg/day. The effect of the dose on the occurrence of serious malformations was not demonstrated by the International Pregnancy Registry, North American AED Pregnancy Registry, Australian Pregnancy Register or Danish Medical Birth Registry [35]. Eline M. P. Poels et al. point out that little is known about the specific impact of lithium on the development of malformations [41]. After reviewing 29 studies, Michele Fornaro et al. found that lithium use during pregnancy was associated with the likelihood of any birth defects and heart defects. The risk of using lithium throughout pregnancy is low. However, the use of lithium in the first trimester and in high doses significantly affected the risk of spontaneous abortion and development of heart defects. In contrast, the risk of malformations was associated with the entire period of pregnancy. Lithium use reduced the risk of postpartum relapse [40]. Concerns associated with the use of mood stabilizers during pregnancy have also been addressed in other works [37,38,39].

### 7.4. Antipsychotics/Neuroleptics

#### 7.4.1. Atypical Neuroleptics/Second-Generation Antipsychotics

The dispute over the effect of atypical neuroleptics and second-generation antipsychotics on the fetus has not been resolved. Unfortunately, there are no clear data confirming or excluding the risk of teratogenicity. It seems reasonable not to use these drugs in the first trimester. During the third trimester, neonates may experience withdrawal symptoms and extrapyramidal symptoms (abnormal muscle movements). This is associated with agitation, increased/decreased muscle tone, tremors, drowsiness, breathing and feeding difficulties. Reported complications include low birth weight and premature birth. Attention should be paid to the risk of using second-generation neuroleptics associated with the development of metabolic syndrome in pregnant women. Vigilance and follow-up are recommended. There is a study showing the effect of using olanzapine and quetiapine on the development of gestational diabetes. Olanzapine may contribute to large neonates due to its metabolic effects on the fetus [36]. There have been reports of studies in animals showing an increased incidence of stillbirths and low birth weights following the use of risperidone during pregnancy. Olanzapine use has been associated with low weight. This phenomenon is dose-dependent. In the long-term postnatal outcomes of olanzapine treatment, no behavioral or cognitive effects have been observed in children of early school age. In many of the studies conducted, no serious malformations were reported as a result of treatment with risperidone or olanzapine [36]. During studies with quetiapine, no association was found between the use of quetiapine during pregnancy and an increased risk of major malformations. In recent reviews, exposure to aripiprazole, quetiapine and olanzapine had no effect on major congenital malformations [10]. Risperidone and paliperidone caused a small increase in the risk of malformations but when used in the third trimester may cause extrapyramidal symptoms in the fetus and neonate. The higher the dose of the neuroleptic, the greater the risk of preterm delivery. The effect of hyperprolactinemia on the fetus is unknown [6,24].

#### 7.4.2. First-Generation Antipsychotics/Classic Neuroleptics

First-generation antipsychotics/classic neuroleptics belong to the best-known group of drugs used in psychiatry and are considered to be substances with a low teratogenic potential; however, they should be avoided in the first trimester if possible. The most common are haloperidol and chlorpromazine. When using classic neuroleptics in the third trimester of pregnancy, there is a risk of withdrawal symptoms (awakening, abnormal muscle tone, tremors, drowsiness, breathing difficulties, feeding problems) or abnormal muscle function. There is a risk of drug-induced parkinsonism, jaundice and increased or decreased neonatal reflexes. According to some reports, the use of haloperidol may have contributed to limb deformities in children [8,42].

#### 7.4.3. A Review of Literature on Antipsychotics

According to Krista F. Huybrechts et al. (2016), the use of antipsychotics in early pregnancy does not significantly increase the risk of congenital malformations, including heart defects. Only with the use of risperidone was a slight increase in the risk of such defects observed. The study examined 1,341,715 eligible pregnancies selected from a cohort of 1,360,101 live births registered in the Medicaid Analytic Extract database [43]. Maria Ellfolk et al. (2021) in a cohort study with 1,273,987 pregnant women included both live births and stillbirths as well as termination of pregnancy due to a severe genetic defect of the fetus. Pregnancies exposed to teratogenic and genetic conditions were excluded. The women were divided into three groups, i.e., exposed to second-generation neuroleptics, exposed to first-generation neuroleptics and not exposed to neuroleptics. They showed that the use of second-generation neuroleptics in early pregnancy is not associated with an increased risk of genetic defects in the fetus. In the group of second-generation neuroleptics, the use of olanzapine was associated with the highest risk of birth defects [44]. Chittaranjan Andrade compared the mega-analysis and two studies that were not included in it [45]. A meta-analysis of data from six observational studies showed that exposure to antipsychotics during pregnancy was not associated with a significantly increased risk of major congenital malformations (MCMs). It has been noted that neither quetiapine nor aripiprazole was associated with an increased risk of MCMs after the first trimester of pregnancy [39]. According to Elizabeth Brunner et al. (2013), in prospective studies, the rates of broad fetal complications identified during pregnancies in which mothers took olanzapine did not differ from those reported in the general population [46]. Important matters associated with the use of neuroleptic during pregnancy have also been addressed by other authors [47,48,49].

### 7.5. Anxiolytics and Benzodiazepines

Taking benzodiazepines increases the risk of birth defects, especially in the first trimester. They affect low birth weight. When taken before birth, they can cause withdrawal symptoms, reduced muscle tone and respiratory depression in the newborn. You can also find information about complications during the use of alprazolam [50]. Bupropion and varenicline should not be prescribed. According to Suzan Uzun et al. (2010), the use of benzodiazepines in pregnancy may contribute to cleft mouth, floppy infant syndrome or pronounced withdrawal symptoms in newborns, based on a review of the literature [4,24,42,51].

### 7.6. Non-Pharmacological Strategies

Due to concerns about pharmacotherapy, women often choose psychological intervention (Table 6). Psychotherapy should be the first-line treatment, preceding pharmacotherapy. According to research, cognitive behavioral therapy (CBT) or interpersonal therapy (IPT) is effective. This applies to individual and group variants. Third-line interventions include neurostimulation, CBT-based attention training, couples therapy, supportive therapy and psychodynamic psychotherapy. Electroconvulsive therapy (ECT) is dedicated to severe, drug-resistant depression with anxiety and suicidal thoughts. Some specialists consider it a first-line treatment due to its safety and effectiveness. Clinicians should consider ECT as a method of choice in many cases of psychiatric patients with contraindications to high-dose pharmacotherapy, such as pregnant female patients. There are reports on the use of repetitive transcranial magnetic stimulation (rTMS), i.e., precranial magnetic stimulation.

## 8. Discussion

It is extremely important to recognize pregnancy-related mood disorders early. The multitude of possible consequences is related to many factors. Psychosis in the mother carries the risk of poorer prenatal care and lack of cooperation during childbirth. After birth, these women may show less interest in children and reluctance to breastfeed. Depressed mothers suffer from their disorders. This might have a very negative psychological impact on the child. In addition, mothers suffering from schizophrenia suffer from affective poverty, which means that the child cannot learn appropriate reactions and interpret them. During an emotional crisis or psychotic episode, there is a risk of a woman committing suicide or an extended suicide. Imperative hallucinations and their delusional interpretations can force a woman to harm her child. Psychiatric patients eat worse and more often smoke cigarettes or reach for psychoactive substances. The poor mental state of a pregnant woman can have a negative impact on the fetus. It is related to epigenetic changes. Much is also said about the influence of endogenous steroids and inflammatory factors. According to the neurodevelopmental theory of depression, biological factors are important, but so is prenatal development throughout the early years of life and into adolescence [3]. Depression in pregnancy increases the risk of low birth weight and premature delivery.

Mental disorders are a global problem affecting various groups of patients. Particularly noteworthy are those related to pregnancy, childbirth and the postpartum period. Often, women put the health of the baby above everything else. They should be made aware that in order for the fetus to be healthy, they must also not become sick and must take care of themselves. Regular mental health assessment with the use of screening tools can reduce the number of negative effects for both mother and baby. Such methods include the Hamilton Depression Rating Scale (HDRS) questionnaire, the Beck Depression Scale and Whooley questions.

Concerns related to pharmacotherapy relate primarily to the effects of drugs on the fetus. Patients often question the effectiveness of the recommended therapy and are afraid of potential addiction, which may result in lack of cooperation in treatment. This can be remedied by conducting appropriate psychoeducation. Both acute psychosis and depression as well as medications can lead to complications. The poor mental state of the mother can be much more harmful than the pharmacotherapy she is taking. Mental disorders are the cause of low birth weight, premature births, etc. A balance of profits and losses should be made, where on the one hand we weigh up the patient’s disease and on the other hand the side effects of drugs. In the case of each patient, attention is paid to an individual approach and joint making of appropriate therapeutic decisions. Psychotherapy should be the first step in the treatment of pregnant women. Next, it is reasonable to reach for pharmacotherapy. The earlier treatment is commenced, the better the prognosis for the patient, the child and their environment.

## 9. Conclusions

Medications contraindicated during pregnancy:

- Paroxetine, fluoxetine (congenital heart defects);
- Clomipramine (malformations of the fetal cardiovascular system);
- Valproates (teratogenic);
- Carbamazepine (e.g., spina bifida);
- Lithium (teratogenic, increases the risk of miscarriage);
- Bupropion (birth defects).

Relatively safe drugs during pregnancy:

- Escitalopram;
- Sertraline;
- Haloperidol;
- Quetiapine.

The lowest effective dose should be used, and researchers draw attention to the correlation between the dose and the toxicity of the drug.

Many reports on the safety of used drugs are contradictory, so it is necessary to explore this topic further.

Non-pharmacological interactions are a good choice. Psychotherapy is of a particular interest. Moreover, in the case of severe conditions and drug resistance, electroconvulsive therapy cannot be ruled out.

Unstable mental state of a pregnant woman may carry a greater risk of complications than pharmacotherapy.

## Figures and Tables

**Table 1:** The ICD-11 psychiatric disorders associated with pregnancy, childbirth and puerperium [1].

Mental or behavioral disorders associated with pregnancy, childbirth or the puerperium Syndromes associated with pregnancy or the puerperium (commencing within about 6 weeks after delivery) that involve significant mental and behavioral features. If the symptoms meet the diagnostic requirements for a specific mental disorder, that diagnosis should also be assigned.	
6E20	Mental or behavioral disorders associated with pregnancy, childbirth or the puerperium, without psychotic symptoms. A syndrome associated with pregnancy or the puerperium (commencing within about 6 weeks after delivery) that involves significant mental and behavioral features, most commonly depressive symptoms. The syndrome does not include delusions, hallucinations or other psychotic symptoms. If the symptoms meet the diagnostic requirements for a specific mental disorder, that diagnosis should also be assigned. This designation should not be used to describe mild and transient depressive symptoms that do not meet the diagnostic requirements for a depressive episode, which may occur soon after delivery (so-called postpartum blues).
6E21	Mental or behavioral disorders associated with pregnancy, childbirth or the puerperium, with psychotic symptoms. A syndrome associated with pregnancy or the puerperium (commencing within about 6 weeks after delivery) that involves significant mental and behavioral features, including delusions, hallucinations or other psychotic symptoms. Mood symptoms (depressive and/or manic) are also typically present. If the symptoms meet the diagnostic requirements for a specific mental disorder, that diagnosis should also be assigned.
6E2Z	Mental or behavioral disorders associated with pregnancy, childbirth or the puerperium, unspecified.
6E40	Psychological or behavioral factors affecting disorders or diseases classified elsewhere. Psychological and behavioral factors affecting disorders or diseases classified elsewhere are those that may adversely affect the manifestation, treatment or course of a condition classified in another chapter of the ICD. These factors may adversely affect the manifestation, treatment or course of the disorder or disease classified in another chapter by interfering with the treatment of the disorder or disease by affecting treatment adherence or care seeking; constituting an additional health risk or influencing the underlying pathophysiology to precipitate or exacerbate symptoms or otherwise necessitate medical attention. This diagnosis should be assigned only when the factors increase the risk of suffering, disability or death and represent a focus of clinical attention and should be assigned together with the diagnosis for the relevant other condition.

**Table 2:** The diagnostic criteria differences [1,10,11].

ICD 11	ICD 10	DSM 5
A brand-new chapter is included in the classification. It includes mental disorders related to pregnancy, childbirth and the postpartum period. It determines whether the main features of the disorder are accompanied by psychotic symptoms or not. In the absence of psychotic symptoms, we most often deal with mood disorders. When psychotic symptoms appear, we diagnose mood disorders and primary psychoses, for example from the schizophrenia group.	No identical chapter to ICD11. The diagnosis of postpartum depression applies only to the puerperium (beginning up to 6 weeks after delivery) and exclusion criteria for other diagnoses. We use the code F53.0: Mild mental and behavioral disorders associated with the puerperium and not otherwise specified: postnatal depression, postpartum depression.	DSM 4: For the first time in the classification, the term “mental disorders with onset after childbirth” is introduced. Postpartum depression develops within 4 weeks of giving birth. DSM 5: In depressive disorders, episodes are distinguished in the perinatal period up to 4 weeks after delivery. When diagnosing psychosis, it should be determined whether it was related to the onset of puerperium up to 4 weeks after delivery.

**Table 3:** Antidepressant safety during pregnancy and breastfeeding.

Publication	Medication	Conclusion
Gao et al., 2017 [21]	fluoxetine	Maternal fluoxetine use is associated with a slightly increased risk of cardiovascular malformations in infants
Kolding et al., 2021 [22]	sertraline	No significant differences in fetal cardiac function in pregnancies exposed to sertraline compared to the unexposed
Bérard A., Zhao J.P., Sheehy O., 2015 [23]	sertraline	Sertraline use during the first trimester of pregnancy was associated with an increased risk of atrial/ventricular defects and craniosynostosis
Huybrechts et al., 2014 [25]	all types of antidepressants	No substantial increase in the risk of cardiac malformations attributable to antidepressant use during the first trimester
Cuomo et al., 2018 [26]	sertraline	Sertraline is one of the safest antidepressants during breastfeeding (limitation: expert opinion only)
Kolding L., Henriksen J.N., Pedersen L.H., 2023 [27]	sertraline	Sertraline is associated with septal heart malformations, but not with more severe heart malformations (limitation: expert opinion only)
Bellantuono et al., 2012 [28]	escitalopram	Escitalopram might be considered safe during pregnancy, in particular as far as major malformations is concerned
Gentile S., 2014 [30]	tricyclic antidepressants (TCAs)	Prenatal clomipramine exposure may increase the risk of cardiac defects. TCA are connected with risk of prenatal antidepressant exposure syndrome. There is a slight increase in safety if TCAs (except clomipramine) are used in late pregnancy
Vitale et al., 2016 [31]	all types of antidepressants	Regarding antidepressants, only paroxetine seems to lead to an increased risk of malformations, whereas fluoxetine, fluvoxamine, sertraline, citalopram, escitalopram and venlafaxine do not appear to increase this risk
Gao S.Y. et al., 2018 [32]	SSRIs	The authors suggest a generally small risk of congenital malformations and argue against a substantial teratogenic effect of SSRIs. Caution is advisable in making decisions about treatment with SSRIs during pregnancy
Womersley K., Ripullone K., Agius M., 2017 [33]	SSRIs	The literature shows that paroxetine and fluoxetine have the strongest association with negative outcomes (significant malformations). The associations between sertraline and citalopram with negative outcomes remain mixed and generally unsubstantiated
Nielsen R.E., Damkier P., 2012 [34]	all types of antidepressants	Citalopram and sertraline can be used during pregnancy, while some controversy remains over in utero exposure to paroxetine and fluoxetine, which might be associated with an increased risk of fetal cardiovascular malformation

**Table 4:** Birth defects caused by valproate use in pregnancy [36].

Birth Defects Caused by Valproate Use in Pregnancy
Neural tube defects (spinal bifida, anencephaly) Craniofacial abnormalities Intrauterine growth retardation Microcephaly Cardiac defects (septal defect) Hypospadias Valproate syndrome: Facial hypoplasia Ceanth wrinkles Short nose with inverted nostrils, flat nasal bridge Long philtrum Long, narrow superior lip, thick lower scale Microstomy, downturned corners of the mouth Hypertelorism Cardiovascular malformations Psychomotor retardation

**Table 5:** The effects of lithium carbonate use depending on the period of pregnancy [36].

First trimester The greatest risk-teratogenic potential. May cause cardiac complications (Ebstein syndrome).
Second and third trimester Risk of reversible hypothyroidism, non-toxic goiter, neurogenic diabetes insipidus or hypoglycemia.
3rd trimester Risk of neonatal adaptation syndrome (floppy infant syndrome). In order to prevent this, lithium administration should be stopped 1–2 days before delivery by caesarean section, returning to it immediately after delivery. Perinatal period. Risk of blood pressure drops in the newborn; if possible, lithium administration should be withheld.

**Table 6:** SIGN, NICE, APA, Beyond Blue recommendations [15,16,17,18].

Mild and Moderate Postpartum Depression	Severe Postpartum Depression
Computer programs based on the assumptions of CBT therapy	CBT or interpersonal psychotherapy
Exercise	Pharmacotherapy if psychotherapy is not sufficient
Psychosocial interventions	
Non-directive counseling (active listening)	
CBT psychotherapy	
Interpersonal psychotherapy	
Pharmacotherapy when other methods fail	

## Data Availability

https://www.ncbi.nlm.nih.gov, accessed on 27 December 2022.
